# The Progress of Active Immunotherapy for Parkinson’s Disease

**DOI:** 10.3390/ijms27052194

**Published:** 2026-02-26

**Authors:** Daniel Busot, Haiqiang Yang, Darrell Sawmiller, Chuanhai Cao

**Affiliations:** 1 Department of Pharmaceutical Sciences, Taneja College of Pharmacy, University of South Florida, Tampa, FL 33612, USA; 2 Department of Chemistry, College of Arts & Sciences, University of South Florida, Tampa, FL 33612, USA; 3 MegaNano BioTech, Inc., 3802 Spectrum Blvd., Suite 122, Tampa, FL 33612, USA; darrel.sawmiller@megananobiotech.com; 4 MegaNano Diagnostics, Inc., 3802 Spectrum Blvd., Suite 149, Tampa, FL 33612, USA; 5 USF Health Byrd Alzheimer’s Institute, University of South Florida, Tampa, FL 33613, USA

**Keywords:** Parkinson’s disease, immunotherapy, vaccine, dendritic cells, antibody, alpha synuclein

## Abstract

Parkinson’s disease (PD) is a multifactorial neurodegenerative disorder defined by nigrostriatal dopaminergic neuron loss and the pathological aggregation of alpha synuclein, yet current clinical interventions remain largely symptomatic and fail to alter long-term disease progression. Emerging evidence demonstrates that immune dysregulation and chronic neuroinflammation contribute significantly to PD pathology, supporting the rationale for active immunotherapy as a disease modifying strategy. This review examines contemporary active immunotherapy platforms including peptide vaccines, genetic vaccination strategies, and antigen sensitized dendritic cell (DC) vaccines with emphasis on the dual capacity of DC based approaches to enhance pathological protein clearance and restore immune homeostasis. Technical limitations and translational barriers are evaluated such as immune heterogeneity among patients, the blood–brain barrier, and variability in DC vaccine manufacturing. Finally, future research directions are proposed including individualized immunologic profiling for treatment stratification, long-acting immunomodulatory formulations, and development of isoform specific DC powder vaccines capable of targeted alpha synuclein engagement. Collectively, these advances highlight active immunotherapy as a promising pathway toward disease modification in Parkinson’s disease.

## 1. Parkinson’s Disease Pathology

Parkinson’s disease (PD) is recognized as a multifactorial neurodegenerative disorder, characterized primarily by the progressive degeneration of dopaminergic neurons in the substantia nigra pars compacta and the intracellular accumulation of misfolded α-synuclein protein [1,2,3]. These pathological alterations result in basal ganglia circuit dysregulation, which underlies the manifestation of cardinal motor symptoms-tremor, muscular rigidity, and bradykinesia-as well as a range of non-motor features, including cognitive impairment and autonomic dysfunction [4,5,6,7]. The etiopathogenesis of PD is understood to involve a complex interplay of genetic, environmental, metabolic, and immunological factors, which can be summarized in Table 1.

A defining molecular feature of PD involves the misfolding and aggregation of α-synuclein into oligomeric and fibrillar structures [18,19], culminating in the formation of Lewy bodies-recognized pathological markers of the disease [20,21,22,23]. These aggregates exert neurotoxic effects by impairing synaptic vesicle trafficking, disrupting neurotransmission, damaging mitochondrial function, and inducing oxidative stress. Moreover, a prion-like propagation mechanism has been proposed, whereby α-synuclein pathology spreads from the lower brainstem (e.g., dorsal motor nucleus of the vagus) to the midbrain and eventually the cerebral cortex, thereby contributing to disease progression [24,25,26].

Sustained activation of microglia and the resultant release of pro-inflammatory cytokines-including tumor necrosis factor-alpha (TNF-α) and interleukin-1 beta (IL-1)-contribute to progressive neuronal degeneration [27].

Current clinical management of Parkinson’s disease focuses primarily on symptomatic relief rather than altering disease progression. The cornerstone of pharmacological therapy is levodopa (often combined with carbidopa), a dopamine precursor that crosses the blood–brain barrier and is converted to dopamine to improve motor symptoms such as bradykinesia and rigidity; it remains the gold standard treatment but is associated with long-term complications such as motor fluctuations and dyskinesias [28]. Adjunctive oral agents, including dopamine agonists, monoamine oxidase-B inhibitors, and catechol-O-methyltransferase inhibitors, are used to enhance dopaminergic signaling or manage motor fluctuations, although these strategies remain symptomatic [28]. For patients with advanced disease or those who develop unpredictable responses to medication, deep brain stimulation is a surgical option in which implanted electrodes deliver electrical pulses to specific brain regions to reduce motor symptoms and allow for lower medication doses [29]. Despite improving quality of life, these approaches remain symptomatic and do not directly target pathogenic protein aggregation or associated immune-mediated mechanisms implicated in Parkinson’s disease. In contrast, immunotherapeutic strategies aim to target disease-relevant pathological proteins and modulate dysregulated immune pathways, offering a potential disease-modifying approach beyond symptomatic control, including active vaccination strategies designed to elicit targeted immune responses.

## 2. Role of α-Synuclein

α-Synuclein (α-Syn) is a highly conserved presynaptic protein that plays a crucial role under physiological conditions by mediating synaptic vesicle trafficking, modulating neurotransmitter release, and maintaining synaptic plasticity [30,31]. It has been demonstrated that α-Syn interacts with phospholipids on vesicular membranes to facilitate vesicle clustering, docking, and exocytosis [32]. α-Syn functions as a molecular chaperone to promote the assembly of the Soluble N-ethylmaleimide-Sensitive Factor (NSF) Attachment Protein Receptors (SNARE) complex, which is essential for membrane fusion [33,34,35]. While α-Syn predominantly exists as an unstructured monomer in the cytosol, its N-terminal (amino-terminal) domain adopts an α-helical structure upon membrane association, enabling functional engagement [36].

In contrast, conformational alterations of α-Syn under pathological conditions—particularly in synucleinopathies such as Parkinson’s disease (PD)-lead to its aggregation from soluble monomers into oligomers and fibrillar intermediates, eventually forming insoluble amyloid fibrils [37,38,39]. These fibrils accumulate into Lewy bodies, the histopathological hallmark of PD. The aggregated forms not only lose their physiological functionality but also acquire cytotoxic properties [40].

Structural and functional analyses have demonstrated that the C-terminal domain of α-syn possesses multiple intrinsic anti-aggregative features, including maintenance of protein solubility via its high net negative charge, formation of protective long-range interactions with the hydrophobic NAC (non-amyloid-β component) domain, and the β-sheet-inhibiting influence of proline residues [41,42,43]. Truncation of this domain abolishes these protective effects, markedly accelerating α-syn aggregation both in vitro and in vivo. Moreover, even substoichiometric amounts of C-terminally truncated α-syn are capable of co-polymerizing with full-length α-syn into fibrils, thereby catalyzing the initiation of pathological aggregation [44,45]. Experimental evidence from biochemical assays, cell cultures, and animal models consistently indicates that C-terminal truncation enhances aggregation propensity and inclusion-forming capacity, in some cases resulting in exacerbated dopaminergic neurodegeneration [46,47].

The “prion-like propagation” hypothesis has received considerable support in recent years. It posits that misfolded α-Syn aggregates propagate across neurons through exosomal transport, synaptic release, or direct membrane penetration [48,49,50]. Upon entering recipient cells, these aggregates serve as pathogenic “seeds” that recruit native α-Syn and induce templated misfolding, thereby facilitating disease progression. Experimental models have corroborated this mechanism, demonstrating that intracerebral injection of pathological α-Syn in mice induces widespread Lewy body-like inclusions [51,52].

Mitochondrial dysfunction is a major axis of α-Syn toxicity. Oligomeric forms target mitochondrial membranes, inhibit complex I of the electron transport chain, and reduce ATP production, leading to elevated ROS levels and oxidative damage [53]. Additionally, pore-forming α-Syn oligomers induce calcium influx and mitochondrial membrane depolarization, triggering apoptosis [54,55].

Neuroinflammation constitutes another critical pathogenic pathway. Extracellular α-Syn aggregates are recognized by microglia via pattern recognition receptors such as Toll-like receptors (TLRs) and inflammasomes like NLRP3, resulting in the release of proinflammatory cytokines including TNF-α and IL-1β [56]. These mediators compromise neuronal integrity, enhance blood–brain barrier permeability, and promote peripheral immune cell infiltration, thereby establishing a chronic inflammatory milieu [57,58]. α-Syn has been implicated in gut–brain axis signaling, whereby it ascends from the enteric nervous system to the brainstem via the vagus nerve—a process potentially triggered by dysbiosis of the gut microbiota and associated immune activation [59].

## 3. Immunological Basis for Parkinson’s Disease

Increasing evidence supports a central role for immune dysregulation in the initiation and progression of PD. While traditionally viewed through the lens of neurotransmitter depletion, recent studies have highlighted the significant contribution of immune dysregulation and chronic neuroinflammation to PD pathogenesis [60]. The immune system, particularly regulatory T cells (Tregs) and microglial activation, plays a critical role in modulating disease progression [61]. Immunotherapies targeting T cell subsets aim to correct the imbalance between proinflammatory and anti-inflammatory immune responses [62]. Tregs, which secrete IL-10 and TGF-β, are essential for suppressing microglial activation and mitigating the neurotoxic actions of effector T cells (Teffs) [63]. In PD, reductions in Treg number and function have been correlated with exacerbated neurodegeneration [62].

Granulocyte-macrophage colony-stimulating factor (GM-CSF) has been shown to promote the differentiation of tolerogenic dendritic cells (DCs), which facilitate Treg expansion. In MPTP-induced mouse models, GM-CSF treatment reduced microglial activation and preserved dopaminergic neurons [64]. A Phase I clinical trial (NCT03790670) demonstrated that sargramostim administration was safe, increased peripheral Treg levels, and improved motor outcomes [65,66]. Similarly, anti-CD3 monoclonal antibodies induce apoptosis or anergy in Teffs and enhance Treg induction via TGF-β-mediated modulation of DCs [67]. While primarily investigated in autoimmune diseases, their potential in PD warrants further investigation. Vasoactive intestinal peptide (VIP) and its receptor agonists can promote the development of tolerogenic DCs and regulatory T cells (Tregs and Tr1), thereby suppressing inflammatory responses [68]. In α-synuclein-overexpressing rat models, VIP analogs such as LBT3627 have shown significant reductions in neuroinflammation and improvements in motor behavior [69].

The aberrant aggregation of α-synuclein contributes not only to intracellular inclusions but also to immune activation. Misfolded α-synuclein is recognized by Toll-like receptors (TLR2/4) and CD36 on glial cells, leading to the release of TNF-α and IL-6 [70,71]. Nitrated α-synuclein can be presented by antigen-presenting cells to T lymphocytes, initiating autoimmune responses [72]. Therapies employing anti-α-synuclein antibodies or vaccines have shown potential in enhancing clearance of pathological aggregates and providing neuroprotection in preclinical studies [60,73]. Small molecule inhibitors and peptides that prevent α-synuclein oligomerization are under investigation to mitigate toxicity [74]. Immune responses directed at α-synuclein can be downregulated through Treg induction or cytokine-based therapies, using agents such as GM-CSF or VIP [75].

Immunotherapy represents a paradigm shift in PD treatment, offering the potential to transition from symptomatic relief to disease modification. Success will depend on a deeper understanding of neuroimmune interactions and the development of innovative, targeted delivery systems.

## 4. The History of Active Immunotherapy for Parkinson’s Disease

Active immunotherapy refers to interventions that stimulate the host immune system to generate an antigen specific adaptive immune response against disease associated targets, most commonly through vaccination-based strategies. This is distinct from passive immunotherapy, which relies on administration of exogenously produced antibodies without inducing immune memory, and from broader immunomodulatory treatments that alter immune signaling or immune cell populations without targeting a defined antigen. Accordingly, immunomodulatory agents such as GM-CSF, VIP analogs, or anti-CD3 antibodies are considered complementary to active immunotherapeutic approaches.

Previous reviews of immunotherapy in Parkinson’s disease have primarily surveyed a broad range of approaches or focused on passive antibody-based strategies. In contrast, this review concentrates on active immunotherapy as a distinct therapeutic category and places particular emphasis on dendritic cell-based vaccination. Rather than viewing these approaches solely as tools for enhancing α-synuclein clearance, this review highlights their potential to also modulate neuroinflammation and restore immune balance. The discussion also considers practical translational factors, including patient-to-patient immune variability, blood–brain barrier constraints, and manufacturing consistency, which are relevant to the clinical development of these therapies. Together, these perspectives support the development of more precise, antigen selective active immunotherapy strategies aimed at disease modification in Parkinson’s disease.

In Alzheimer’s disease, a landmark study demonstrated that immunization of transgenic mice with Aβ42 prevented β-amyloid plaque formation, neuritic dystrophy, and astrogliosis, providing early proof-of-concept for pathology-modifying immunotherapy in the central nervous system [76]. However, early Alzheimer’s active-vaccine trials demonstrated safety risk, including meningoencephalitis in ~6% of participants [77]. Consequently, subsequent efforts shifted toward elucidating the underlying mechanisms of immune-mediated neurotoxicity and refining active immunotherapy approaches to achieve therapeutic efficacy without inducing detrimental inflammatory responses, particularly in the nervous system, which lacks the regenerative capacity of many peripheral tissues [78]. The application of immunotherapeutic strategies to Parkinson’s disease (PD) was initially delayed by the prevailing view that such approaches would be most effective in disorders characterized by abundantly extracellular pathological proteins, such as amyloid-β in Alzheimer’s disease [79]. However, accumulating evidence demonstrating that α-synuclein can be released into the extracellular space and participate in intercellular propagation renewed interest in immunotherapy for PD [80]. A major concern in developing vaccines against endogenous proteins is the risk of inducing autoimmune responses. However, because pathological α-synuclein exists in structurally distinct aggregated conformations, vaccine strategies can be designed to preferentially target these toxic isoforms while minimizing immune recognition of the native monomeric protein. As a result, PD has experienced a growing number of preclinical and clinical immunotherapy studies, with particular optimism surrounding antibody-based approaches designed to target extracellular pathological protein species [81]. In parallel, other disease-modifying strategies, including regenerative cell-based therapies and protein clearance approaches, have been explored in PD but have thus far failed to demonstrate sustained long-term clinical benefit [82]. Active immunotherapy possesses several attributes that make it an attractive therapeutic strategy for Parkinson’s disease. By engaging the host immune system, active immunotherapy induces immune memory, resulting in a long-lasting immune response, analogous to prophylactic vaccination, which generates sustained humoral immunity with elevated antibody titers. Consequently, endogenous antibody production elicited by active immunization may reduce the need for frequent dosing when compared with passive immunotherapy approaches that rely on repeated antibody administration [77]. Although disease-modifying efficacy has not yet been demonstrated for active immunotherapy in Parkinson’s disease, such effects have been observed in other conditions.

Several strategies have been explored to address Parkinson’s disease (PD) through active immunotherapy. Peptide-based therapeutic vaccines are designed to mimic epitopes of disease-associated proteins, thereby eliciting a targeted immune response [83]. In PD, the peptide vaccines PD01A and PD03 have demonstrated promise in mechanistic clinical studies by inducing antibody responses against misfolded α-synuclein [84]. Notably, PD01A induced a detectable α-synuclein–specific antibody response in 89% of treated patients [84]. Beyond antigen-specific vaccination approaches, active immunotherapy strategies in PD have also explored immune modulation through targeted manipulation of T-cell responses. In a 1-methyl-4-phenyl-1,2,3,6-tetrahydropyridine (MPTP) mouse model, adoptive transfer of naturally occurring regulatory T cells attenuated Th17-mediated nigrostriatal dopaminergic neurodegeneration [85].

Although these strategies represent valid approaches to active immunotherapy, each is associated with important limitations. Peptide-based therapeutic vaccines are inherently restricted by epitope specificity and may fail to capture the structural heterogeneity of misfolded α-synuclein species present in Parkinson’s disease. In addition, these vaccines primarily bias immune responses toward humoral antibody production, offering limited control over the magnitude or quality of cellular immune engagement. T cell–directed immunomodulatory approaches raise concerns regarding the breadth of immune activity and the risk of excessive immune suppression, particularly in older patient populations with altered immune homeostasis. Genetic vaccination strategies, including viral vector– and DNA plasmid–based platforms, introduce additional challenges related to variable antigen expression, inconsistent immune activation, and the potential induction of autoreactive immune responses. Dendritic cell vaccines may address several of these limitations by facilitating antigen-specific immune activation with greater control over immune engagement. Accordingly, DC vaccines represent a promising active immunotherapy platform and are discussed in greater detail in the next section. An overview of the major α-synuclein-targeted active immunotherapy platforms discussed in this review, including their antigen targets, delivery strategies, mechanisms of immune action, and stages of clinical development, is provided in Table 2.

For the ongoing study ACI-7104.056 (NCT06015841), comprehensive safety conclusions cannot yet be drawn, as peer-reviewed clinical data are not currently available and formal safety assessments remain in progress. In a first-in-human phase 1 study of the UBITh-based peptide vaccine UB-312, active immunization against α-synuclein was primarily associated with mild, transient local and systemic adverse events, with no evidence of treatment-related neuroinflammation, clinically significant T-cell activation, or sustained inflammatory cytokine responses, although higher-dose cohorts were halted following isolated flu-like reactions [86]. In phase 1 studies of PD01A and PD03A in patients with early Parkinson’s disease, safety profiles were similarly dominated by transient injection-site reactions and infrequent mild systemic symptoms, with no evidence of immune-mediated neuroinflammation, meningoencephalitis, treatment-related serious adverse events, or MRI or laboratory findings suggestive of CNS inflammatory toxicity during follow-up [87,88]. Beyond clinical studies, preclinical evaluations of MultiTEP-based DNA and recombinant protein vaccines assessed safety through histological, immunological, and neuropathological analyses in transgenic mouse models, which did not reveal overt evidence of vaccine-associated neuroinflammatory changes or immune-mediated tissue pathology within the scope of the analyses performed [89].

Although preclinical and early clinical studies support the feasibility of active immunotherapy targeting α-synuclein, most clinical investigations to date have been limited to early phase trials focused on safety and immunogenicity rather than clinical efficacy. Preclinical models have provided important mechanistic insight but may not fully recapitulate the complexity, progression, or immune heterogeneity of Parkinson’s disease in humans. These limitations highlight the need for cautious interpretation of existing data and for future studies designed to rigorously assess disease modifying outcomes. Overall, current clinical data support the feasibility and immunogenic potential of active immunotherapy in Parkinson’s disease but remain insufficient to establish clear therapeutic or disease-modifying efficacy.

Peptide vaccines, genetic vaccination strategies, and dendritic cell-based vaccines are all discussed as components of active immunotherapy in Parkinson’s disease. Greater emphasis is placed on dendritic cell-based approaches due to their broader mechanistic scope, including both antigen specific clearance and immune regulatory functions, as well as their potential relevance for precision immunotherapy. Peptide and genetic vaccination strategies are therefore presented more concisely to contextualize current clinical progress and limitations.

## 5. Antigen-Sensitized DC as Vaccines for PD

Dendritic cell (DC) vaccine therapy represents a precision immunotherapy strategy grounded in the principle of antigen-presenting cell (APC)-mediated immune activation [90]. Sipuleucel-T, the first FDA-approved autologous APC-based immunotherapy, provides clinical proof that personalized cellular vaccines are feasible and safe in humans [91]. However, the reliance on patient-derived autologous cells introduces challenges related to manufacturing consistency, scalability, and functional variability, which remain key barriers to broader clinical implementation. Basically, DCs can be differentiated into two subtypes, immature and mature, upon the exposure to inflammatory agents. This approach involves the ex vivo differentiation of patient-derived monocytes into immature DCs under the stimulation of granulocyte-macrophage colony-stimulating factor (GM-CSF) and interleukin-4 (IL-4) [92]. Subsequent antigen loading is achieved through tumor lysates, synthetic peptide fragments, or mRNA encoding neoantigens, followed by maturation induced by toll-like receptor (TLR) agonists-such as lipopolysaccharide (LPS) or polyinosinic-polycytidylic acid or proinflammatory cytokines, including IL-1β and interferon-γ (IFN-γ) [93,94,95]. These mature DCs, characterized by high expression of CD83 and HLA-DR, are reinfused into the host, where they migrate to secondary lymphoid tissues to initiate antigen-specific T cell responses via major histocompatibility complex (MHC) class I and II presentation to CD8^+^ and CD4^+^ T cells, respectively, along with critical costimulatory signals (e.g., CD80/CD86-CD28 and CD40-CD40L) to promote polyclonal T cell activation [96,97,98].

Dendritic cell (DC)-based vaccination has emerged as a promising immunotherapeutic approach with unique potential in the treatment of neurodegenerative disorders. The therapeutic efficacy of DC vaccines is primarily attributed to their dual immunomodulatory capacity: first, through the activation of antigen-specific T cells, they facilitate the clearance of pathological proteins; second, through the generation of tolerogenic DCs (tolDCs), they mitigate neuroinflammatory responses [99,100]. In Alzheimer’s disease (AD), the second-generation Aβ-targeted vaccine CAD106 demonstrated favorable safety profiles-avoiding meningoencephalitis-and modest therapeutic benefits, including a 15–20% reduction in cerebrospinal fluid (CSF) Aβ42 levels during Phase II clinical trials [101]. Similarly, the Tau-targeted vaccine AADvac1, in a Phase III study, significantly slowed the rate of neurofilament light chain (NfL) elevation by 35% and reduced the annual decline in Mini-Mental State Examination (MMSE) scores by 0.5–1.0 points [102]. 

Despite these advances, the clinical application of DC vaccines in neurodegenerative diseases continues to face several technical hurdles. These include limited efficiency in traversing the blood–brain barrier (BBB)—with current intranasal delivery methods achieving only 0.1–0.5% migration rates—challenges in epitope selection that must balance immunogenicity with safety, and the labor-intensive, highly variable nature of individualized vaccine manufacturing (with traditional protocols showing batch variation exceeding 40% [103,104,105,106]. Recent technological progress has offered potential solutions: genetic modification of DCs to overexpress CXCR4 has increased their adhesion to brain microvascular endothelial cells by up to eightfold; in silico epitope prediction tools such as NetMHC-4.0 have reduced the time required for epitope screening by approximately 70% [107,108]; and automated production platforms such as CliniMACS Prodigy have streamlined vaccine generation, reducing processing time from 14 to 5 days and improving inter-batch consistency to within 15% [109,110].

From a translational perspective, several DC vaccine candidates have advanced into various stages of clinical development, including ACI-24 (Phase II, AD), PD01A (Phase I/II, PD), and NP001 (Phase II, amyotrophic lateral sclerosis) [87,111,112].

## 6. Dead vs. Live DC Vaccines

Dendritic cell (DC) vaccines can be broadly categorized into live DC vaccines and non-viable (dead) DC vaccines. Live DC vaccines consist of functional, viable dendritic cells that are ex vivo antigen-loaded and then reinfused into the patient [113]. Once administered, these cells retain their full biological activity: they process and present antigens through both MHC class I and II pathways, leading to activation of CD8^+^ cytotoxic T cells and CD4^+^ helper T cells [114]. Live DCs can also migrate to lymph nodes, secrete cytokines, and contribute to the development of immunological memory [115]. Clinical studies have demonstrated promising outcomes using this approach, including improved overall survival in patients treated with DC based immunotherapies [116]. However, several limitations remain, including complex cell-culture requirements, challenges in maintaining cell viability, and higher costs due to the individualized nature of the therapy.

In contrast, dead DC vaccines utilize dendritic cells that have been fixed or otherwise rendered non-viable after antigen loading. Although these cells are incapable of migration, cytokine secretion, or directly priming T cells, they still present antigen on their surface and can be internalized by host dendritic cells through cross-presentation [117]. This strategy offers advantages in terms of manufacturing, storage, and distribution, and would be less expensive than live DC vaccines. Nonetheless, DC preparations that are unable to actively process and present antigen instead rely on cross-presentation by the patient’s endogenous dendritic cells, which may lead to more variable immune activation [118,119].

Despite their therapeutic promise, dendritic cell–based vaccines present translational challenges related to manufacturing consistency and scalability. Autologous DC production requires patient-specific cell isolation, ex vivo differentiation, antigen loading, and quality control, which can introduce variability across batches and increase production complexity. In addition, the labor- and infrastructure-intensive nature of DC manufacturing may limit scalability compared with more standardized vaccine platforms. These considerations underscore the importance of continued optimization of DC manufacturing strategies alongside evaluation of clinical efficacy.

## 7. Future Directions

Current therapies for Parkinson’s disease provide symptomatic benefit but do not alter the underlying neurodegenerative process and are often associated with long-term complications or diminishing efficacy over time. These limitations underscore the need for disease-modifying strategies, motivating continued investigation of immunotherapeutic approaches targeting pathogenic mechanisms such as α-synuclein aggregation.

Patient-to-patient differences in immune status are likely to influence the feasibility and consistency of active immunotherapy as a disease-modifying approach in Parkinson’s disease. Variability in baseline inflammation, regulatory immune function, and antigen responsiveness may limit the effectiveness of uniform vaccine strategies, highlighting the importance of patient stratification and immune profiling in future therapeutic development. Continued development of active immunotherapies for Parkinson’s disease (PD) must account for the immune variability that naturally exists among patients. A single standardized dosing or vaccine approach will likely be inadequate and may pose unintended harm in immunocompromised or autoimmune-prone patients [120,121]. Advancing this field requires streamlined screening methods capable of stratifying patients into treatment-appropriate categories based on immune phenotype, Treg-to-Teffector ratios, and markers of neuroinflammation [122,123]. Investigations should evaluate how these metrics correlate with clinical outcomes, and which patient groups respond most favorably to specific active immunotherapy modalities.

Despite encouraging data, several barriers to clinical translation remain. The restricted permeability of the BBB limits CNS access of many immunomodulatory agents [124,125]. As a result, the efficacy of active immunotherapy largely depends on achieving sufficient peripheral antibody titers and leveraging Fc-mediated transport and microglial engagement to influence extracellular or propagating α-synuclein species within the CNS. Potential solutions include the use of nanocarriers (e.g., liposomal encapsulation of GM-CSF) and focused ultrasound-mediated drug delivery [126,127]. Systemic immune modulation carries inherent risks of infection or autoimmune sequelae [128]. Therefore, strategies that enable CNS-specific or conditionally activated immune interventions are essential. Given the immunophenotypic heterogeneity among PD patients, precision immunotherapeutic approaches guided by patient-specific biomarkers-such as peripheral Treg/Teff ratios-are necessary to optimize treatment efficacy.

Although vaccines are traditionally prophylactic, greater focus must shift toward therapeutic vaccines designed to enhance endogenous immune function in the presence of active pathology [84]. For PD, active immunotherapy is needed to disrupt α-synuclein propagation, enhance the clearance of pathological protein aggregates, and restore immune homeostasis in neuroinflammatory niches of the substantia nigra and basal ganglia [21].

Greater mechanistic research is also needed to understand and improve the efficacy of DC-based vaccines. Isoform-specific DC powder vaccines represent one promising avenue [129]. By selectively presenting pathogenic α-synuclein isoforms while leaving physiologic forms untouched, such vaccines could enhance therapeutic precision and reduce the likelihood of autoimmune responses. Powder-based formulations may also improve vaccine stability, minimize reliance on cold-chain storage, reduce manufacturing costs, and lower batch-to-batch variability, enabling partial standardization without compromising patient-specific adaptation [130]. Future optimization may involve the incorporation of multi-epitope antigens to broaden immune coverage, as well as the use of advanced delivery systems such as nanoparticle carriers to enhance antigen stability and targeted delivery. By combining multiple antigens or multiple conformational epitopes of α-synuclein, these platforms could activate a broader and more durable T-cell response [131]. Such an approach would reduce the likelihood that pathological α-synuclein species evade immune detection [132].

Notably, combination strategies have yielded enhanced therapeutic outcomes: co-administration of DC vaccines with PD-1 inhibitors has resulted in a threefold increase in T cell infiltration in AD models, while combinatorial treatment with glial cell line-derived neurotrophic factor (GDNF) has promoted dopaminergic neuron survival [133,134]. Key areas for innovation include the development of multi-epitope vaccine platforms, the design of universal DC vaccines utilizing HLA-transgenic lines such as DCOne^®^, and the engineering of intelligent DC systems responsive to inflammatory cues through promoters sensitive to transcription factors like NF-κB [135]. Accordingly, future strategies may improve feasibility by incorporating epitope selection approaches designed to engage multiple HLA allele types, thereby enhancing cross-population immunogenic coverage. These emerging technologies are expected to significantly advance the clinical translation of DC vaccines for neurodegenerative diseases within the next decade.

Integrating this approach with multi-omics analyses and advanced neuroimaging tools (e.g., PET tracers for α-syn) will deepen mechanistic understanding and accelerate the development of personalized treatment strategies for synucleinopathy. Future research priorities should focus on improving the pharmacokinetics of immunomodulatory agents (e.g., long-acting formulations of GM-CSF or VIP analogs), investigating the application of gene editing technologies such as CAR-Tregs in neurodegenerative diseases, and conducting large-scale, controlled clinical trials to establish the long-term efficacy and safety of immune-based interventions. But there are several challenges remaining, including the restrictive nature of the BBB, insufficient antigenic specificity of current interventions, limitations inherent to preclinical animal models, and unresolved safety concerns. Despite these obstacles, advancements in delivery technologies and precision-targeting approaches have demonstrated promising potential for clinical translation.

Among the future directions discussed, approaches most likely to have near-term clinical impact include optimization of peptide-based vaccines, improved patient stratification using immune or biomarker profiles, and refinement of dosing and scheduling within existing vaccine platforms already in clinical development. In contrast, strategies such as advanced BBB-modulation technologies, highly personalized dendritic cell-based vaccines, and next-generation delivery systems represent promising but less mature approaches, given their manufacturing, regulatory, and scalability challenges. Together, these considerations highlight a distinction between incremental advances that can be tested in upcoming trials and more exploratory approaches requiring substantial further development.

## Figures and Tables

**Table 1:** Pathogenetic factors of PD.

Pathological Factor Category	Specific Examples	Proposed Pathological Mechanism	Representative References
Protein-related factors	α-synuclein aggregation, Lewy bodies	Protein misfolding, impaired proteostasis, neuronal toxicity	[8,9]
Metal exposure	Iron, copper	Transition metals like Fe^2+^ and Cu^2+^ facilitate aberrant cross-linking via redox reactions	[10,11,12]
Pesticides and environmental toxins	Rotenone, 1-methyl-4-phenyl-1,2,3,6-tetrahydropyridine (MPTP)	Mitochondrial complex I inhibition, dopaminergic neuron toxicity	[13]
Genetic factors	*PRKN*, *PINK1*, Parkin, DJ-1, *SNCA*, *LRRK2*	*PINK1* and Parkin mutations impair mitochondrial quality control, while *SNCA* mutations, gene multiplications, or promoter variants increase α-synuclein expression and aggregation, promoting Parkinsonian pathology.	[14,15,16]
Other contributing factors	Oxidative stress, neuroinflammation, mitochondrial dysfunction	Progressive dopaminergic neuron loss	[17]

**Table 2:** Summary of α-Synuclein targeted vaccine platforms in development.

Vaccine/Platform	Antigen Target	Delivery Platform	Mechanism of Immune Action	Clinical Stage	Outcome	Adjuvant
ACI-7104.056 (VacSYn) NCT06015841	Pathological aggregated α-synuclein	Adjuvanted protein–peptide conjugate vaccine (intramuscular)	Intended induction of α-synuclein–specific immune responses	Phase 2 ongoing	Phase 2 ongoing	Yes
UB-312 [86]	10-residue C-terminal α-synuclein epitope	Synthetic α-syn peptide conjugated to a proprietary UBITh T-helper peptide	Induction of α-syn-specific IgG antibodies targeting pathological α-syn species via UBITh-mediated CD4^+^ T-helper support	Phase 1 completed	Safe, strong IgG responses, CSF antibody detection	Yes
PD01A (AFFITOPE) [87]	C-terminal α-synuclein epitope mimic (8–amino acid peptide)	KLH-conjugated short synthetic peptide vaccine, adsorbed to aluminum hydroxide	KLH-conjugated α-syn B-cell epitope vaccine inducing humoral IgG responses against oligomeric α-syn	Phase 1 completed	Safe, immunogenic, moderate antibody durability	Yes
PD03A (AFFITOPE) [88]	C-terminal α-synuclein epitope mimic (10–amino acid peptide)	KLH-conjugated short synthetic peptide vaccine, adsorbed to aluminum hydroxide	Induction of α-synuclein–specific IgG antibodies targeting pathological α-syn species via a short B-cell epitope peptide conjugated to KLH, providing T-helper support	Phase 1 completed	Safe, immunogenic but lower titers vs. PD01A	Yes
PV-1950R (MultiTEP Recombinant Vaccine) [89]	Three α-synuclein B-cell epitopes (aa 85–99, 109–126, 126–140)	Recombinant MultiTEP protein vaccine	Recombinant protein presenting three α-synuclein B-cell epitopes within the MultiTEP scaffold, inducing robust humoral IgG responses supported by MultiTEP-driven CD4^+^ T-helper activation	Preclinical	Robust antibody responses, motor and pathology improvement	No

## Data Availability

No new data were created or analyzed in this study. Data sharing is not applicable to this article.
